# Association of dietary inflammatory index and oxidative balance score with all-cause and cardiovascular mortality in US non-diabetic adults

**DOI:** 10.3389/fnut.2025.1607162

**Published:** 2025-08-22

**Authors:** YuNan Han, Lin Li, YongXiang Wang, Wen Fan

**Affiliations:** ^1^Department of Endocrinology, The First Affiliated Hospital of Yangtze University, Jingzhou, Hubei, China; ^2^Department of Medicine, Yangtze University, Jingzhou, Hubei, China

**Keywords:** dietary inflammatory index, dietary oxidative balance score, all-cause death, cardiovascular mortality, non-diabetic patients, NHANES

## Abstract

**Background:**

Inflammation and oxidative stress (OS) are critical factors in the pathogenesis of chronic diseases (CDs), with dietary factors being a central modifiable determinant. This study aimed to assess the association of the Dietary Inflammation Index (DII) and Dietary Oxidative Balance Score (DOBS) with all-cause and cardiovascular (CV) mortality in non-diabetic adults.

**Methods:**

Data on non-diabetic adults were extracted from the National Health and Nutrition Examination Survey (NHANES) (2009–2018). Dietary information was collected via 24-h recalls, and DII and DOBS were calculated. Multivariate weighted Cox proportional hazards models, Kaplan–Meier (KM) survival analysis, and restricted cubic spline (RCS) analyses were conducted to assess mortality associations. Subgroup analyses were performed based on gender, age, BMI, smoking status, hypertension, and hyperlipidemia.

**Results:**

After applying multivariable–weighted Cox proportional hazards regression, participants with the highest DII quartile exhibited elevated risks of all-cause mortality [Q4: HR = 1.554 (1.258, 1.934)] and CV mortality [Q4: HR = 2.100 (1.307, 3.375)]. In contrast, the highest DOBS quartile was linked to reduced all-cause mortality [Q4: HR = 0.724 (0.553, 0.946)], with no significant association observed for CV mortality. RCS analyses confirmed a positive dose–response between DII and both mortality outcomes, as well as an inverse relationship for DOBS. Subgroup analyses revealed that high DOBS (Q4) scores were negatively associated with all-cause and CV mortality in women, individuals aged ≥60 years, current smokers, hypertensive individuals, and those without dyslipidemia. High DII (Q4) scores were positively associated with all-cause mortality across all sexes, individuals aged ≥60 years, smokers, and those with hypertension or dyslipidemia. Additionally, high DII scores were associated with CV mortality among women, both smokers and non-smokers, and individuals without hypertension or dyslipidemia.

**Conclusion:**

Higher DOBS levels are associated with lower all-cause mortality, while higher DII levels are linked to increased all-cause and CV mortality. Dietary interventions targeting inflammation may reduce mortality risks, thereby informing public health strategies.

## 1 Introduction

Inflammation and oxidative stress (OS) are identified as physiological responses to external stimuli and endogenous damage. Moreover, they play essential roles in maintaining immune homeostasis and the repair process (1). However, chronic inflammation and OS at elevated levels not only disrupt organismal homeostasis but are also considered primary drivers of various chronic diseases, including cardiovascular diseases (CVDs), cancer, and neurodegenerative disorders (2, 3). Numerous studies have shown that biological markers of inflammation and OS (such as C-reactive protein [CRP] and redox imbalance) are closely associated with disease progression and risk of death (4, 5). Therefore, to prevent chronic diseases, it is now crucial to identify the key factors regulating inflammation and OS levels.

The dietary inflammatory index (DII) and the dietary oxidative balance score (DOBS) are two key indicators to quantify the effects of diets on inflammation and OS, respectively. These two indicators are used to evaluate the pro-inflammatory/anti-inflammatory potential and the pro-oxidative/antioxidant capacity of diets (6, 7). DOBS and DII have been shown to be associated with the mortality and incidence of CVDs in patients with diabetes and prediabetes. Each unit increase in DOBS was associated with a 1.8% reduction in all-cause mortality and a 4% reduction in cardiovascular mortality among patients with diabetes and prediabetes (8). DOBS is also inversely associated with the incidence of chronic kidney disease (9), hypertension (10), depression (11), and other conditions. Meanwhile, in patients with diabetes and prediabetes, a high DII is more strongly associated with the risk of CVDs (12). Additionally, a prospective cohort study revealed that DOBS and DII were significantly associated with all-cause, CVDs, and cancer mortality (13). Thus, it is evident that existing research primarily focuses on individuals with diabetes or metabolic disorders, as well as the general population. However, patients with diabetes often exhibit metabolic disturbances such as insulin resistance, hyperglycemia, and dyslipidemia. These factors, by promoting chronic inflammation and OS, complicate the mechanistic pathways through which dietary factors exert their effects (14). Notably, although natural antioxidants (e.g., polyphenols) can effectively scavenge reactive oxygen species (ROS), the chronic inflammatory milieu in diabetes persistently suppresses the activity of antioxidant enzymes (such as superoxide dismutase [SOD] and glutathione peroxidase [GPx]), thereby significantly diminishing the efficacy of exogenous antioxidants. For example, quercetin exerts potent effects in healthy models but typically requires higher doses to achieve comparable efficacy in diabetic models. This inflammatory microenvironment suppresses the Nrf2 pathway, diminishes endogenous antioxidant defenses, and elevates the threshold at which dietary antioxidants can exert their protective effects [39674630]. Moreover, these metabolic abnormalities not only drive diabetes progression but may also interfere with nutrient metabolism and utilization (15), thereby modulating the independent effects of dietary factors on disease progression and mortality risk. In other words, among individuals with metabolic dysfunction, it is difficult to discern whether the observed increase in mortality risk is attributable to intrinsic metabolic derangements or dietary intake patterns. Conversely, the specific metabolic disorders mentioned above were not observed in non-diabetic patients. Within this group, the levels of inflammation and OS are more likely to be directly influenced by dietary variables and less likely to be confused by metabolic disorders associated with diabetes. To better elucidate the causal relationship between diet and health outcomes, the independent associations of DII and DOBS with the risk of death should be assessed in a non-diabetic population.

However, the association of DOBS and DII with the risk of death in non-diabetic patients has not been previously examined. To address this research gap, the present study utilized data from the National Health and Nutrition Examination Survey (NHANES) to investigate the association of DOBS and DII with both all-cause mortality and cardiovascular mortality in non-diabetic patients. We hypothesized that, in non-diabetic individuals, a higher DII is associated with increased all-cause and cardiovascular mortality, whereas a higher DOBS is associated with decreased all-cause and cardiovascular mortality.

## 2 Data and methods

### 2.1 Study participants

**Figure 1:**
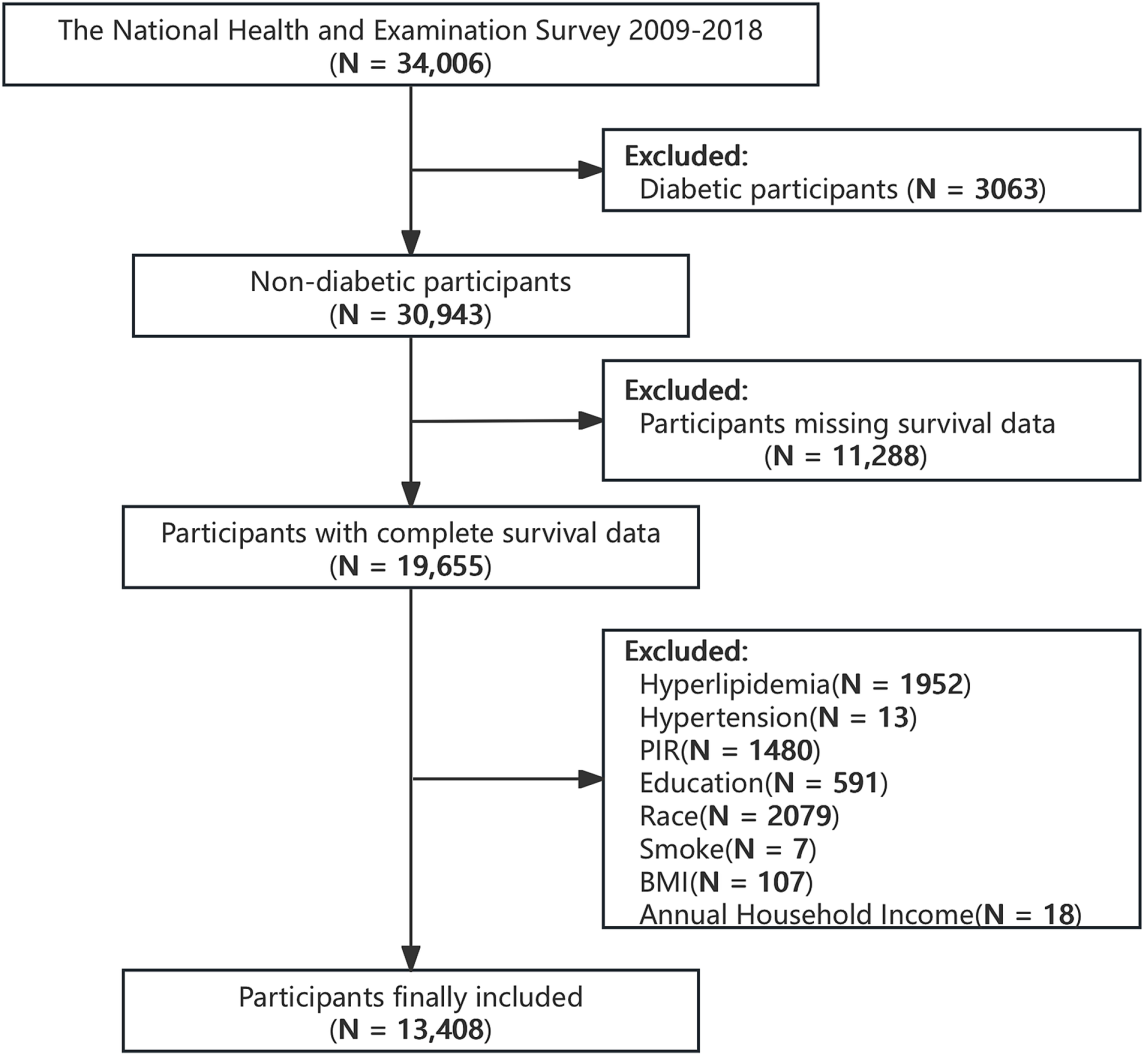
Flowchart of the selection strategy.

The NHANES is an ongoing survey performed in the United States by the CDC using a stratified, multi-stage sampling design. When linked with follow-up mortality data, it functions as a widely utilized, nationally representative prospective cohort study (16). Data from 2009 to 2018 were used in this research, involving individuals aged 18 years or older. Diabetes was defined as a self-reported physician diagnosis of fasting blood glucose (FBG) ≥ 7 mmol/L, glycosylated hemoglobin (HbA1c) ≥ 6.5%, or current use of hypoglycemic medications (17). We excluded participants who met the definition of diabetes (*N* = 3,123), those with missing covariate data (hyperlipidemia: *N* = 1,952, hypertension: *N* = 13, poverty: *N* = 1,480, educational level: *N* = 591, ethnicity: *N* = 2,079, smoking history: *N* = 7, annual family income: *N* = 18, body mass index (BMI): *N* = 107), and those with missing death data (*N* = 45). After excluding the aforementioned individuals, a total of 13,408 patients were included in the final research (Figure 1).

The NHANES obtained approval from the National Center for Health Statistics Research Ethics Review Board and was performed in accordance with the ethical standards as laid down in the 1964 Declaration of Helsinki and its later amendments or comparable ethical standards. Each participant gave a written informed consent agreement when they were enrolled in the NHANES, and the National Center for Health Statistics’ ethics review board approved the study.

### 2.2 Dietary data

The assessment of daily dietary intake was performed through 24-h dietary recall interviews conducted on 2 consecutive days. Each dietary nutrient and the overall dietary energy were computed following the guidelines outlined in the USDA’s Food and Nutrient Database for Dietary Studies (FNDDS) (18). Information on dietary supplement use was collected through specialized questionnaires. In addition, the nutrient assessment did not include dietary components derived from dietary supplements.

### 2.3 Assessment of DOBS

The DOBS was calculated by scoring the combination of pro-oxidant and antioxidant nutrients in the diet (7). Drawing on previous insights regarding the connection between certain nutrients and DOBS, 16 nutrients were comprehensively evaluated, including 2 pro-oxidants and 14 antioxidants (19). The nutritional data were collected from two 24-h dietary reviews. The continuous dietary components were categorized into tertiles, with antioxidant scores ranging from 1 to 3 and pro-oxidant scores assigned in the opposite manner. Each participant’s dietary intake level was determined by summing the scores assigned to all dietary components. The detailed composition of DOBS components is provided in Supplementary Table 1.

### 2.4 Assessment of DII

The dietary inflammatory index (DII) is a dietary tool designed to assess the overall pro- and anti-inflammatory properties of an individual’s diet. It incorporates 45 dietary components identified from the scientific literature and selected based on their effects on inflammatory indicators, including CRP, interleukin-6 (IL-6), and tumor necrosis factor alpha (TNF-*α*). The scoring scale for each component ranges from −1 (anti-inflammatory) to +1 (pro-inflammatory) (20). The Food Frequency Questionnaire (FFQ) or 24-h dietary recall was applied to assess participants’ dietary intake. Considering the differences in energy intake, the intake was standardized per 1,000 kcal. Subsequently, the intake was adjusted using the formula [(daily intake - global average daily intake) divided by the standard deviation of the global average daily intake]. The modified intake values were then multiplied by the cumulative inflammatory response score of each dietary component (21). The total DII scores for each participant were calculated, with a higher score indicating a greater consumption of pro-inflammatory foods. Information on DII dietary components is detailed in Supplementary Table 2.

### 2.5 Determination of mortality

Within our research, the main outcome parameters encompassed all-cause mortality and cardiovascular (CV) mortality, as ascertained through the ICD-10 classification system [International Classification of Diseases (ICD), 10th edition]. The classification codes for CV deaths were I00–I09, I11, I13, and I20–I51. More detailed information is available at https://www.cdc.gov/nchs/linked-data/mortality-files/?CDC_AAref_Val=https://www.cdc.gov/nchs/data-linkage/mortality.htm.

### 2.6 Covariate assessment

The covariates included in this study were gender, age, ethnicity, smoking status, BMI, hypertension, hyperlipidemia, educational level, and poverty–income ratio (PIR). Among these covariates, demographic data were collected through family interviews. Smoking was defined as having smoked at least 100 cigarettes in one’s lifetime. BMI was calculated as weight divided by height squared (kg/m^2^). Hypertension was defined as a systolic blood pressure greater than or equal to 130 mmHg and a diastolic blood pressure greater than or equal to 80 mmHg. Hyperlipidemia was defined as LDL-C levels ≥ 4.9 mmol/L (22). Educational attainment was categorized as high school or lower, high school graduates, some college, or college or higher. PIR was applied as an indicator of poverty status, calculated as the total household income divided by the poverty threshold. According to the guidelines, PIR was classified into three groups: ≤1.3, 1.3–3.5, and >3.5 (23).

### 2.7 Statistical analysis

All analyses in this study adhered to NHANES analysis guidelines. We applied the multi-stage sampling design by incorporating the main sampling units, pseudo variances, and masked variances in sampling weights to ensure nationally representative estimates. Given the intricate sampling structure of NHANES, a 2-day dietary sample was chosen following appropriate weighting procedures (1/5 * WTDR2D). Median values were used to represent continuous variables (P25, P75), whereas categorical variables were depicted using numbers (percentages). In this study, the comparison of categorical variables between groups was conducted using Pearson’s chi-square test. Since the continuous variables in this research did not follow a normal distribution according to the Kolmogorov–Smirnov test, non-parametric tests were used to assess group differences.

DII and DOBS were categorized into quartiles for statistical analysis purposes (taking the lowest quartile Q1 as the reference group). A multivariable weighted Cox regression model with restricted cubic spline (RCS) analysis was utilized to estimate hazard ratios (HRs) and 95% confidence intervals (CIs), assessing the correlation of DII and DOBS with all-cause and CV death in non-diabetic patients. Adjustments were made based on gender, age, BMI, ethnicity, smoking status, hypertension, hyperlipidemia, and educational level. Log-rank tests and Kaplan–Meier (KM) survival analyses were performed to examine the differences in survival rates according to DOBS and DII in the non-diabetic population. Subsequently, subgroup analyses were performed to explore the potential modifying impacts of key demographic and clinical variables on the associations between DII, DOBS, and mortality outcomes. All covariates (excluding those used for stratification) were adjusted in the model. These analyses were stratified based on gender (male/female), smoking status (yes/no), presence of hypertension (yes/no), and hyperlipidemia (yes/no). The interaction of DII and DOBS with these variables was assessed by incorporating interaction terms into the Cox proportional hazards regression model. Specifically, multiplicative interaction terms (DII × covariate and DOBS × covariate) were included to assess whether the association between dietary scores and mortality differed across subgroups. Analysis of variance was performed to assess the significance of interactions. The propensity score matching (PSM) was utilized to mitigate potential selection bias and confounding variables. A logistic regression model incorporating relevant covariates (gender, age, ethnicity/nationality, educational level, BMI, smoking status, hypertension, and hyperlipidemia) was applied to estimate propensity scores. Based on these propensity scores, a 1:2 nearest-neighbor matching algorithm was adopted to pair participants. The balance after matching was assessed by comparing standardized mean differences (SMDs) of covariates between matched groups, with SMD < 0.1 indicating an acceptable balance. Subsequently, a weighted Cox proportional hazards regression model was used in the matched cohort to evaluate the associations of DII and DOBS with mortality outcomes, aiming to minimize potential bias and ensure the reliability of the study results. Finally, participants with prediabetes and those receiving insulin therapy were excluded, and the primary analyses were re-conducted to assess the robustness of the findings. Statistical analyses were carried out using R 4.3.0 (R Foundation for Statistical Computing, Vienna, Austria), with statistical significance defined as a *p*-value of < 0.05.

## 3 Results

### 3.1 Baseline characteristics

A total of 13,408 non-diabetic patients aged over 18 years were enrolled. Table 1 displays the baseline characteristics of participants stratified by all-cause mortality and survival status. The median age of the cohort was 46 years, with 53% of participants being female (*N* = 7,207) and 74% identifying as non-Hispanic White (*N* = 6,843). This study examined differences in variables including gender, age, ethnicity, educational level, family income, PIR, smoking status, hypertension, hyperlipidemia, and BMI between deceased and surviving participants (*p* < 0.05).

**Table 1:** Baseline information from the death and survival groups.

Characteristic	Overall, *N* = 13,408^1^	Death, *N* = 12,511^1^	Survival, *N* = 897^1^	*p*-value^2^
Gender				<0.001
Male (%)	6,201 (47%)	5,691 (47%)	510 (56%)	
Female (%)	7,207 (53%)	6,820 (53%)	387 (44%)	
Age, years	46 (33, 59)	45 (32, 58)	70 (57, 80)	<0.001
Ethnicity				<0.001
Mexican American (%)	1,892 (8.6%)	1,834 (8.9%)	58 (3.4%)	
Other Hispanic (%)	1,468 (6.0%)	1,430 (6.2%)	38 (2.0%)	
Non-Hispanic White (%)	6,843 (74%)	6,208 (74%)	635 (86%)	
Non-Hispanic Black (%)	3,205 (11%)	3,039 (11%)	166 (8.9%)	
Education level				<0.001
Below high school	2,590 (12%)	2,349 (12%)	241 (19%)	
High school	3,151 (23%)	2,905 (22%)	246 (28%)	
Some college	4,384 (32%)	4,139 (33%)	245 (29%)	
College graduate or above	3,283 (33%)	3,118 (33%)	165 (24%)	
Annual household income	9.0 (6.0,15.0)	9.0 (6.0,15.0)	7.0 (5.0,10.0)	<0.001
Poverty income ratio	3.15 (1.53,5.00)	3.23 (1.57,5.00)	2.23 (1.22,4.25)	<0.001
Smoking status	5,879 (42%)	5,330 (42%)	549 (60%)	<0.001
BMI, kg/m^2^	28 (24, 33)	28 (24, 33)	27 (24, 32)	0.3
Hypertension (%)	4,518 (29%)	3,940 (27%)	578 (60%)	<0.001
Hyperlipidemia (%)	4,404 (32%)	3,979 (31%)	425 (47%)	<0.001

Continuous variables were represented by weighted mean (SE). Categorical variables were expressed in the form of counts (weighted percentages).

^1^Median (25, 75%); n (unweighted) (%); ^2^Wilcoxon rank-sum test for complex survey samples; chi-square test with Rao–Scott’s second-order correction.

### 3.2 Associations of DOBS and DII with all-cause and cardiovascular mortality

**Figure 2:**
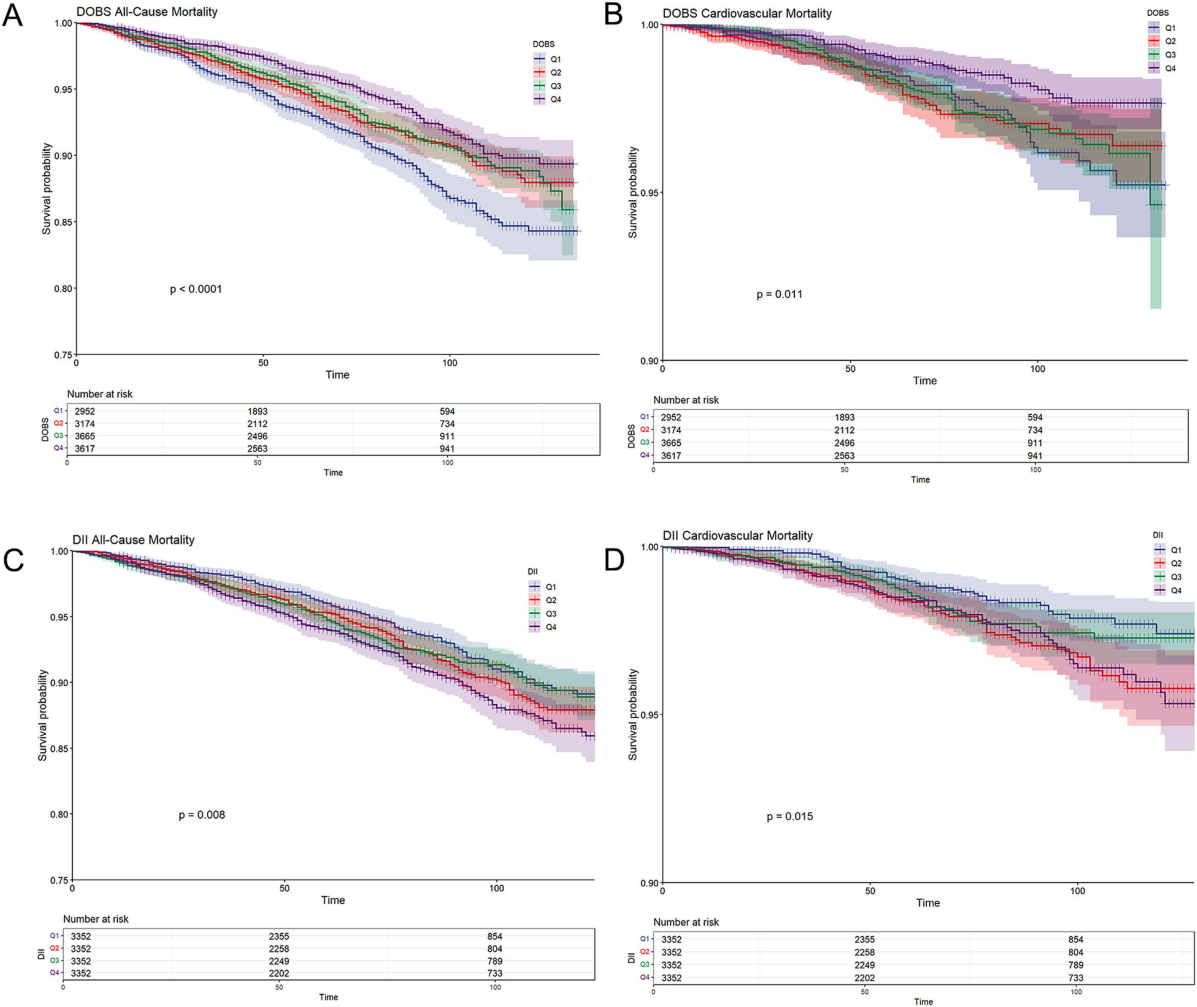
**(A)** Kaplan–Meier survival curves for all-cause mortality stratified by dietary oxidative balance score (DOBS) quartiles; **(B)** Kaplan–Meier survival curves for cardiovascular mortality stratified by DOBS quartile; **(C)** Kaplan–Meier survival curves for all-cause mortality stratified by dietary inflammatory index (DII) quartiles; and **(D)** Kaplan–Meier survival curves for cardiovascular mortality stratified by DII quartile. Non-diabetic participants were grouped into quartiles based on cohort-specific distributions of DOBS and DII. Survival probabilities (solid lines) and 95% confidence intervals (shaded bands) were estimated using the Kaplan–Meier method, and between-group differences were assessed using the log-rank test.

Tables 2, 3 present the correlation of DOBS and DII with all-cause mortality and CV mortality. It could be concluded from Table 2 that in both the rough and adjusted models, DOBS showed a negative association with all-cause mortality (*p* < 0.05), whereas DII showed a positive association with all-cause mortality (*p* < 0.01). Specifically, after adjusting for all covariates, individuals in the highest quartile (Q4) of DOBS exhibited a 27.6% lower risk of all-cause mortality in comparison to those in the lowest quartile (Q1) [HR = 0.724 (95%CI: 0.553, 0.946)], whereas individuals in the highest quartile (Q4) of DII showed a 55.4% higher risk of all-cause mortality [HR = 1.554 (95%CI: 1.248, 1.934)]. It could be observed from Table 3 that DII had a positive association with CV mortality in both the rough and adjusted models, while DOBS was not significantly associated with CV mortality in participants (*p* > 0.05). Individuals in the highest quartile (Q4) of DII showed a 110% higher risk of CV death [HR = 2.100 (95%CI: 1.307, 3.375)] in comparison to those in the lowest quartile (Q1). Participants in the highest quartile (Q4) of DOBS exhibited the lowest risk of all-cause and cardiovascular mortality, whereas those in the lowest quartile (Q1) showed the highest risk (log-rank *p* < 0.0001; log-rank *p* = 0.011, respectively) (Figures 2A,B). In contrast, participants in the highest quartile (Q4) of DII experienced the highest risk of all-cause and cardiovascular mortality, while those in the lowest quartile (Q1) had the lowest risk (log-rank *p* = 0.008; log-rank *p* = 0.015, respectively) (Figures 2C,D).

**Table 2:** Correlation of DOBS and DII with all-cause mortality.

All-cause mortality	Model 1		Model 2	
	HR (95%CI)	*p*	HR (95%CI)	*p*
DOBS				
Q1	—	—	—	—
Q2	0.750 (0.591, 0.953)	0.018	0.837 (0.662, 1.057)	0.135
Q3	0.722 (0.564, 0.925)	0.01	0.793 (0.608, 1.035)	0.088
Q4	0.642 (0.486, 0.850)	0.002	0.724 (0.553, 0.946)	0.018
DII				
Q1	—	—	—	—
Q2	1.345 (1.020, 1.772)	0.035	1.372 (1.052, 1.790)	0.02
Q3	1.158 (0.886, 1.520)	0.286	1.254 (0.975, 1.613)	0.078
Q4	1.510 (1.189, 1.918)	<0.001	1.554 (1.248, 1.934)	<0.001

The data were represented by weighted HR estimates and 95%CIs. Model 1 was a rough model, and the adjustment factors in Model 2 included gender, age, ethnicity, BMI, educational level, smoking status, hypertension, and hyperlipidemia.

Model 1: No covariates were adjusted.

Model 2: Gender, age, ethnicity, education, PIR, smoking status, BMI, hypertension, and hyperlipidemia were adjusted.

**Table 3:** Correlation of DOBS and DII with CV mortality.

Cardiovascular mortality	Model 1		Model 2	
	HR (95%CI)	*p*	HR (95%CI)	*p*
DOBS				
Q1	—	—	—	—
Q2	1.239 (0.827, 1.858)	0.299	1.397 (0.919, 2.124)	0.118
Q3	0.953 (0.636, 1.428)	0.816	1.073 (0.705, 1.633)	0.742
Q4	0.707 (0.398, 1.257)	0.238	0.837 (0.458, 1.532)	0.565
DII				
Q1	—	—	—	—
Q2	2.228 (1.332, 3.729)	0.002	2.206 (1.288, 3.779)	0.004
Q3	1.494 (0.897, 2.488)	0.123	1.599 (0.944, 2.709)	0.081
Q4	2.046 (1.334, 3.139)	0.001	2.100 (1.307, 3.375)	0.002

The data were represented by weighted HR estimates and 95%CIs. Model 1 was a rough model, and the adjustment factors in Model 2 included gender, age, ethnicity, BMI, educational level, smoking status, hypertension, and hyperlipidemia.

Model 1: No covariates were adjusted.

Model 2: Gender, age, ethnicity, education, PIR, smoking status, BMI, hypertension, and hyperlipidemia were adjusted.

### 3.3 Analysis of restricted cubic spline regression

**Figure 3:**
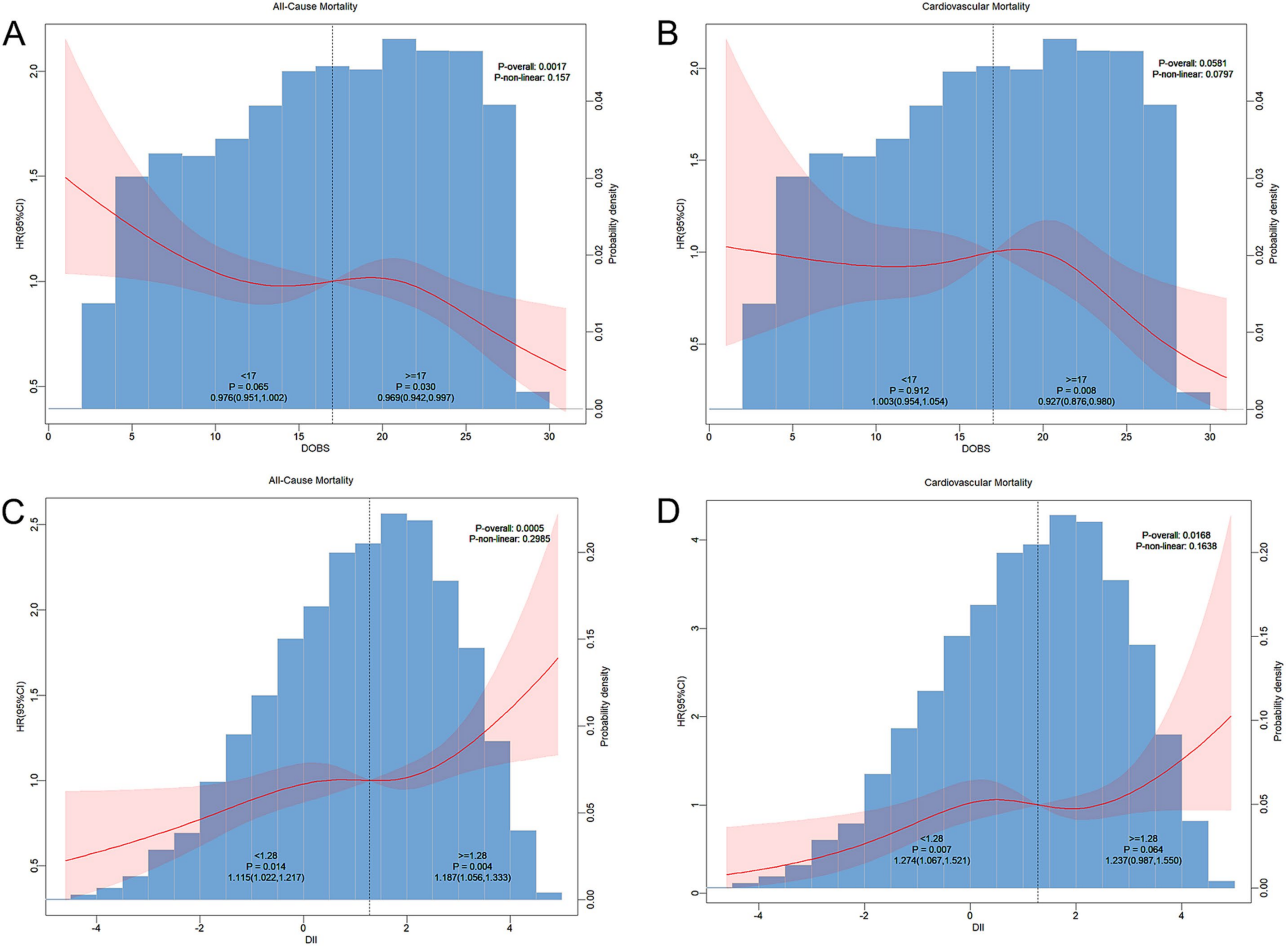
**(A)** RCS curve depicting the association of dietary oxidative balance score (DOBS) with all-cause mortality in non-diabetic participants; **(B)** DOBS with cardiovascular mortality; **(C)** dietary inflammatory index (DII) with all-cause mortality; and **(D)** DII with cardiovascular mortality. Adjustments were made based on gender, age, ethnicity, BMI, educational level, smoking status, hypertension, and hyperlipidemia. The central estimate was represented by a red solid line, and the red shaded area indicated the 95%CI.

Multivariate-adjusted RCS plots revealed a non-linear association of DOBS and DII with all-cause and CV deaths in non-diabetic participants. As shown in Figure 3, DOBS showed a linearly negative correlation with all-cause mortality (*p*-non-linear: 0.157) and CV mortality (*p*-non-linear: 0.0797) in non-diabetic patients. Furthermore, DII showed a linearly positive correlation with all-cause mortality (*p*-non-linear: 0.2985) and CV mortality (*p*-non-linear: 0.1638) in non-diabetic patients.

### 3.4 Subgroup analysis

**Figure 4:**
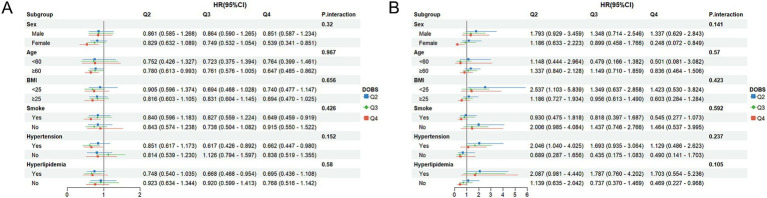
**(A)** Subgroup analysis of OBS and all-cause mortality; **(B)** Subgroup analysis of OBS and CV mortality. The adjustment factors included gender, age, ethnicity, BMI, educational level, smoking status, hypertension, and hyperlipidemia.

**Figure 5:**
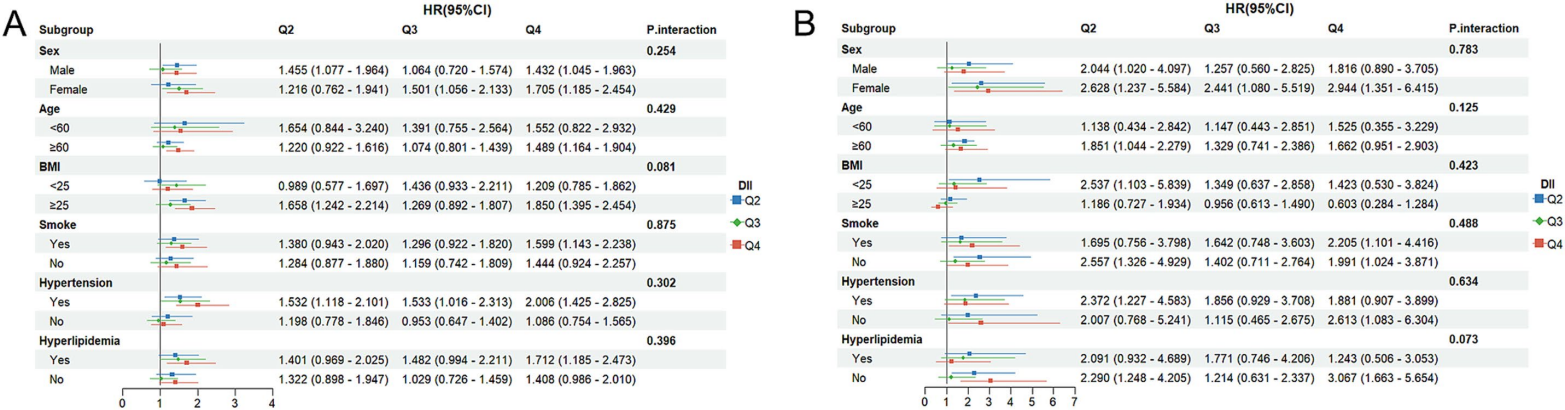
**(A)** Subgroup analysis of DII and all-cause mortality; **(B)** Subgroup analysis of DII and CV mortality. The adjustment factors included gender, age, ethnicity, BMI, educational level, smoking status, hypertension, and hyperlipidemia.

To assess whether the associations of DOBS and DII with mortality differ across subgroups, subgroup analyses were performed. Participants were initially classified based on gender, age, BMI, smoking status, hypertension, and hyperlipidemia. Subsequently, further regression model analysis was conducted on these subgroups. All covariates were adjusted in the model, except for those used for stratification. As shown in Figure 4A, high levels of DOBS (Q4) were significantly negatively associated with all-cause mortality in females (HR = 0.539, 95%CI: 0.341–0.851), individuals aged ≥60 (HR = 0.647, 95%CI: 0.485–0.862), smokers (HR = 0.649, 95%CI: 0.459–0.919), and individuals with hypertension (HR = 0.662, 95%CI: 0.447–0.980). In addition, as shown in Figure 4B, high levels of DOBS (Q4) were significantly negatively associated with CV mortality in females (HR = 0.248, 95%CI: 0.072–0.849) and individuals without hyperlipidemia (HR = 0.469, 95%CI: 0.227–0.968). As shown in Figure 5A, high levels of DII (Q4) showed an obviously positive association with all-cause mortality in males (HR = 1.432, 95%CI: 1.045–1.963), females (HR = 1.705, 95%CI: 1.185–2.454), those aged ≥60 (HR = 1.489, 95%CI: 1.164–1.904), individuals with BMI ≥ 25 (HR = 1.850, 95%CI: 1.395–2.454), smokers (HR = 1.599, 95%CI: 1.143–2.238), individuals with high blood pressure (HR = 2.006, 95%CI: 1.425–2.825), and individuals with hyperlipidemia (HR = 1.712, 95%CI: 1.185–2.473) (*p* < 0.05). Figure 5B illustrates a notable positive correlation between elevated levels of DII (Q4) and CV mortality specifically among females (HR = 2.944, 95%CI: 1.351–6.415), smokers (HR = 2.205, 95%CI: 1.101–4.416), non-smokers (HR = 1.991, 95%CI: 1.024–3.871), and individuals without hypertension (HR = 2.613, 95%CI: 1.083–6.304) or hyperlipidemia (HR = 3.067, 95%CI: 1.664–5.654) (*p* < 0.05). There was no significant interaction between the stratified variables of DOBS and DII subgroups in relation to all-cause and CV death, as indicated by a non-significant *p*-value for interaction (*p* > 0.05).

### 3.5 Sensitivity analysis

To mitigate the influence of confounding variables, we performed a PSM analysis, applied a multivariate Cox risk regression model, and adjusted for various potential confounders in the original (unmatched) cohort. The results were similar to the main estimates reported. The matched results showed that, as shown in Supplementary Table 3, individuals in the highest quartile (Q4) of OBS showed a 28.3% lower risk of all-cause mortality [HR = 0.717 (95%CI: 0.537, 0.956)] in comparison to those in the lowest quartile (Q1). Individuals in the highest quartile (Q4) of DII showed a 55.4% higher risk of all-cause mortality [HR = 1.554 (95%CI: 1.253, 1.928)]. Supplementary Table 4 illustrates no marked association between OBS and CV mortality among participants (*p* > 0.05). Individuals in the highest quartile (Q4) of DII showed a 110% lower risk of CV mortality [HR = 2.100 (95%CI: 1.307, 3.375)] in comparison to those in the lowest quartile (Q1). Furthermore, the potential influence of prediabetes and insulin therapy on the study outcomes was evaluated. To ensure robustness, participants with prediabetes or receiving insulin therapy (*N* = 1,127) were excluded, and the primary analyses were repeated. The effect estimates remained consistent with the main findings (Supplementary Table 5).

## 4 Discussion

By incorporating 45 dietary components associated with inflammation and 16 specific pro-inflammatory and antioxidant nutrients, 2 scoring systems were developed to investigate dietary inflammatory potential and antioxidant nutrient levels in individuals. The research findings suggested that a higher level of DOBS was significantly correlated with a lower risk of all-cause mortality, whereas a higher level of DII was significantly correlated with a higher risk of all-cause and CV mortality. Moreover, DOBS showed a linearly negative correlation with all-cause and CV mortality, while DII showed a linearly positive correlation with all-cause and CV mortality.

Previous studies have demonstrated that elevated DII scores are closely associated with increased risks of obesity, type 2 diabetes mellitus, and cardiovascular disease (24, 25). This relationship is hypothesized to arise because higher DII scores reflect greater consumption of proinflammatory dietary components—such as saturated fats, trans fats, and refined sugars—which have been shown to activate nuclear factor κB (NF-κB) and other inflammatory signaling pathways, thereby promoting systemic inflammation (26, 27). Moreover, increased consumption of these components has been linked to elevated levels of inflammatory biomarkers, including C-reactive protein (CRP) and interleukin-6 (IL-6) (28, 29). These mediators are thought to promote endothelial activation and monocyte adhesion, thereby contributing to arterial injury, atherosclerosis progression, and a heightened risk of cardiovascular events. While prior investigations focused predominantly on diabetic or metabolic syndrome populations, the present study is the first to extend the evaluation of DII in a non-diabetic cohort, revealing similar proinflammatory effects. This finding carries important clinical implications, suggesting that even individuals with relatively healthy metabolic profiles may experience subclinical inflammation induced by a persistently proinflammatory diet, thereby adversely affecting long-term cardiovascular health and increasing all-cause mortality risk.

For instance, in metabolically healthy cohorts, adherence to anti-inflammatory dietary patterns has been associated with reduced oxidative stress and lower levels of inflammatory markers, whereas proinflammatory diets have been correlated with increased inflammation and an elevated risk of metabolic abnormalities (30). Even in non-diabetic individuals with normal glycemic metabolism, chronic exposure to a high-DII diet may induce a subclinical low-grade inflammatory state that eludes routine clinical detection but nonetheless progressively impairs endothelial function and accelerates atherogenesis (31). This chronic inflammation may influence cardiovascular health through multiple pathways, such as affecting insulin signaling or exacerbating oxidative stress. Even if it is insufficient to cause diabetes, it is still capable of inflicting cumulative damage on the vascular system (32).

In addition, a study on the Food Inflammation Index (FII) indicated that the FII performs excellently in predicting individual inflammation levels (such as high-sensitivity C-reactive protein) and is highly correlated with the DII model. It further revealed significant pro- or anti-inflammatory heterogeneity within different food groups, and this heterogeneity may affect the applicability of inflammation indices to specific dietary patterns or populations. For example, mackerel and cod exhibited markedly different anti-inflammatory effects due to differences in their DHA and EPA content (33). This phenomenon suggests that, among populations with different metabolic states or dietary compositions, the effects of the DII may vary. In the current study, the pro-inflammatory effect of a high DII in a non-diabetic cohort may be partly attributed to variations in dietary components. The heterogeneity revealed by the FII within food groups implies that future research may enhance the predictive power of DII in specific populations by incorporating more detailed food classifications, thereby enabling a more accurate elucidation of its mechanisms.

On this basis, another important finding of the present study was that higher Dietary Oxidative Balance Scores (DOBS) were significantly associated with lower all-cause mortality in the non-diabetic population. This finding highlighted the critical role of dietary antioxidant capacity in maintaining health within this specific cohort. In a prospective cohort of 24,527 U.S. participants, Wang et al. (13) reported a significant inverse association between DOBS and all-cause mortality in the general population, but no significant relationship with cardiovascular diseases (CVDs) or cancer mortality was observed. Since the general population includes a substantial proportion of non-diabetic individuals whose metabolic and inflammatory profiles may align more closely with those of our study cohort, Wang’s findings partially support our results. Conversely, the Seguimiento Universidad de Navarra (SUN) cohort study (34) found a strong inverse association between DOBS and cardiovascular mortality (35). This discrepancy may stem from differences in study population characteristics. The SUN cohort consisted of highly educated, middle-aged Spanish adults who adhered to a Mediterranean dietary pattern, potentially enhancing the protective effects of DOBS through their diet and lifestyle. Furthermore, a German cohort of 2,125 diabetic patients demonstrated that elevated oxidative stress biomarkers were significantly associated with both major cardiovascular events and all-cause mortality (36), suggesting that redox imbalance may be a key driver of mortality in diabetic populations. Thus, in contrast to the German study’s diabetic subjects, our focus on non-diabetic individuals may explain why the observed inverse relationship between DOBS and all-cause mortality reflects a lower degree of redox imbalance and fewer metabolic disturbances.

The study has the following advantages. First, our findings are more broadly applicable and generalizable, as they are based on data from a nationally representative, high-quality sample. Second, higher statistical power was embodied in larger sample sizes, longer follow-up periods, as well as stable and reliable outcomes. Third, a more comprehensive understanding of the possible association between diet-related variables and mortality in non-diabetic individuals can be gained by assessing OS and inflammatory responses using two composite indicators, DOBS and DII, rather than a single measure. However, certain limitations are inevitable in this study. First, our study design was observational, precluding causal inferences. Future research could consider Mendelian randomization to explore causality. Second, our dietary data were based on participants’ self-reports, which may be subject to recall and reporting biases. Third, although multiple covariates were adjusted, residual confounding may still exist. Finally, our study included only non-diabetic individuals and did not exclude those with prediabetes, which may limit the generalizability of the findings to fully metabolically healthy populations and hinder the precise assessment of dietary effects across differing health statuses.

## 5 Conclusion

Our research findings indicate that higher DOBS scores are significantly associated with lower all-cause mortality, whereas higher DII scores are significantly associated with higher all-cause mortality. For CV mortality, higher DII scores are significantly correlated with lower mortality rates, while no significant association was observed between DOBS and CV mortality. These findings suggest that improving dietary quality—particularly by reducing the consumption of pro-inflammatory dietary components and increasing the intake of antioxidant-rich foods—may effectively lower inflammation and oxidative stress levels, thereby reducing mortality risk.

## Data Availability

The datasets presented in this study can be found in online repositories. The names of the repository/repositories and accession number(s) can be found in the article/Supplementary material.
