# Supplementary material for: Association of dietary inflammatory index and oxidative balance score with all-cause and cardiovascular mortality in US non-diabetic adults
Source: Front Nutr. 2025 Aug 22;12:1607162. doi: 10.3389/fnut.2025.1607162 (PMC12411158; doi:10.3389/fnut.2025.1607162)
Supplement: Supplementary file 1 [file Table_1.docx]

**Supplementary Materials**

**Supplementary Table 1** Allocation scheme for oxidative balance score

| DOBS components | Property | Scoring assignment | | |
| --- | --- | --- | --- | --- |
|  |  | 0 | 1 | 2 |
| Dietary components | | | | |
| Dietary fiber (g/d) | Antioxidant | Tertile 1 | Tertile 2 | Tertile 3 |
| Carotene (RE/d) | Antioxidant | Tertile 1 | Tertile 2 | Tertile 3 |
| Riboflavin (mg/d) | Antioxidant | Tertile 1 | Tertile 2 | Tertile 3 |
| Niacin (mg/d) | Antioxidant | Tertile 1 | Tertile 2 | Tertile 3 |
| Vitamin B6 (mg/d) | Antioxidant | Tertile 1 | Tertile 2 | Tertile 3 |
| Total folate (mcg/d) | Antioxidant | Tertile 1 | Tertile 2 | Tertile 3 |
| Vitamin B12 (mcg/d) | Antioxidant | Tertile 1 | Tertile 2 | Tertile 3 |
| Vitamin C (mg/d) | Antioxidant | Tertile 1 | Tertile 2 | Tertile 3 |
| Vitamin E (ATE) (mg/d) | Antioxidant | Tertile 1 | Tertile 2 | Tertile 3 |
| Calcium (mg/d) | Antioxidant | Tertile 1 | Tertile 2 | Tertile 3 |
| Magnesium (mg/d) | Antioxidant | Tertile 1 | Tertile 2 | Tertile 3 |
| Zinc (mg/d) | Antioxidant | Tertile 1 | Tertile 2 | Tertile 3 |
| Copper (mg/d) | Antioxidant | Tertile 1 | Tertile 2 | Tertile 3 |
| Selenium (mcg/d) | Antioxidant | Tertile 1 | Tertile 2 | Tertile 3 |
| Total fat (g/d) | Pro-oxidant | Tertile 3 | Tertile 2 | Tertile 1 |
| Iron (mg/d) | Pro-oxidant | Tertile 3 | Tertile 2 | Tertile 1 |

OBS, Oxidative Balance Score; RE, retinol equivalent; ATE, alpha-tocopherol equivalent.

**Supplementary Table 2** Dietary composition parameters involved in DII,inflammatory effect scores, and intake values from the global composite data setc

| Dietary composition parameter | Overall inflammatory effect score^b^ | Global daily mean intake(units/d) | Standard deviation of the global daily intake |
| --- | --- | --- | --- |
| Alcohol (g) | -0.278 | 13.98 | 3.72 |
| Vitamin B12 (μg) | 0.106 | 5.15 | 2.7 |
| Vitamin B6 (mg) | -0.365 | 1.47 | 0.74 |
| β-Carotene (μg) | -0.584 | 3718 | 1720 |
| Caffeine (g) | -0.11 | 8.05 | 6.67 |
| Carbohydrate (g) | 0.097 | 272.2 | 40 |
| Cholesterol (mg) | 0.11 | 279.4 | 51.2 |
| Energy (kcal) | 0.18 | 2056 | 338 |
| Total fat (g) | 0.298 | 71.4 | 19.4 |
| Fiber (g) | -0.663 | 18.8 | 4.9 |
| Folic acid (μg) | -0.19 | 273 | 70.7 |
| Iron (mg) | 0.032 | 13.35 | 3.71 |
| Magnesium (mg) | -0.484 | 310.1 | 139.4 |
| MUFA^d^(g) | -0.009 | 27 | 6.1 |
| Niacin (mg) | -0.246 | 25.9 | 11.77 |
| Protein (g) | 0.021 | 79.4 | 13.9 |
| PUFA^e^(g) | -0.337 | 13.88 | 3.76 |
| Vitamin B2 (mg) | -0.068 | 1.7 | 0.79 |
| Saturated fat (g) | 0.373 | 28.6 | 8 |
| Selenium (μg) | -0.191 | 67 | 25.1 |
| Vitamin B1 (mg) | -0.098 | 1.7 | 0.66 |
| Vitamin A (RE ^a^) | -0.401 | 983.9 | 518.6 |
| Vitamin C (mg) | -0.424 | 118.2 | 43.46 |
| Vitamin D (μg) | -0.446 | 6.26 | 2.21 |
| Vitamin E (mg) | -0.419 | 8.73 | 1.49 |
| Zinc (mg) | -0.313 | 9.84 | 2.19 |

^a^Retinol equivalents.

^b^Dietary composition parameter-specific overall inflammatory effect score.

^c^DII of a certain dietary component = (Daily intake of the dietary component - Global daily mean intake of the dietary component) / Standard deviation of the global daily intake for the dietary component * Overall inflammatory effect score of the dietary component. The DII for each participant was obtained by summing the DII of the 26 dietary components selected in this study.

^d^Monounsaturated fatty acids.

^e^Polyunsaturated fatty acids.

**Supplementary Table 3** Cox regression results of DOBS and DII with all-cause mortality after sensitivity analysis

| All-Cause Mortality | Model | |  |
| --- | --- | --- | --- |
|  | HR(95%CI) | P |  |
| DOBS |  |  |  |
| Q1 | —— | —— |  |
| Q2 | 0.904(0.701,1.167) | 0.44 |  |
| Q3 | 0.732(0.565,0.947) | 0.017 |  |
| Q4 | 0.717(0.537,0.956) | 0.023 |  |
| DII |  |  |  |
| Q1 | —— | —— |  |
| Q2 | 1.280(0.980,1.670) | 0.07 |  |
| Q3 | 1.275(0.969,1.676) | 0.082 |  |
| Q4 | 1.554(1.253,1.928) | <0.001 |  |

Model:Gender, age, race, education, PIR, somke, BMI, hypertension, hyperlipidemia were adjusted.

**Supplementary Table 4** Cox regression results of DOBS and DII with CV mortality after sensitivity analysis

| Cardiovascular Mortality | Model | |
| --- | --- | --- |
|  | HR(95%CI) | P |
| DOBS |  |  |
| Q1 | —— | —— |
| Q2 | 1.397(0.919,2.124) | 0.118 |
| Q3 | 1.073(0.705,1.633) | 0.742 |
| Q4 | 0.837(0.458,1.532) | 0.565 |
| DII |  |  |
| Q1 | —— | —— |
| Q2 | 2.206(1.288,3.779) | 0.004 |
| Q3 | 1.599(0.944,2.709) | 0.081 |
| Q4 | 2.100(1.307,3.375) | 0.002 |

Model:Gender, age, race, education, PIR, somke, BMI, hypertension, hyperlipidemia were adjusted.

**Supplementary Table 5** Cox regression analysis of the associations of DOBS and DII with all-cause and cardiovascular mortality after excluding participants with prediabetes or on insulin therapy in sensitivity analyses.

| ALL-Cause Mortality | Model1 |  | Model2 |  |
| --- | --- | --- | --- | --- |
|  | HR(95%CI) | P | HR(95%CI) | P |
| DOBS |  |  |  |  |
| Q1 | —— | —— | —— | —— |
| Q2 | 0.806(0.620,1.048) | 0.107 | 0.923(0.711,1.198) | 0.545 |
| Q3 | 0.809(0.628,1.043) | 0.102 | 0.885(0.661,1.184) | 0.41 |
| Q4 | 0.643(0.473,0.874) | 0.005 | 0.718(0.531,0.973) | 0.032 |
| DII |  |  |  |  |
| Q1 | —— | —— | —— | —— |
| Q2 | 1.330(1.010,1.753) | 0.043 | 1.359(1.047,1.764) | 0.021 |
| Q3 | 1.135(0.850,1.516) | 0.392 | 1.263(0.975,1.634) | 0.077 |
| Q4 | 1.437(1.111,1.859) | 0.006 | 1.471(1.156,1.871) | 0.002 |
| Cardiovascular Mortality | Model1 |  | Model2 |  |
|  | HR(95%CI) | P | HR(95%CI) | P |
| DOBS |  |  |  |  |
| Q1 | —— | —— | —— | —— |
| Q2 | 1.189(0.766,1.846) | 0.44 | 1.455(0.897,2.359) | 0.129 |
| Q3 | 0.984(0.652,1.485) | 0.939 | 1.141(0.729,1.785) | 0.563 |
| Q4 | 0.510(0.275,0.945) | 0.032 | 0.621(0.321,1.202) | 0.157 |
| DII |  |  |  |  |
| Q1 | —— | —— | —— | —— |
| Q2 | 2.102(1.259,3.510) | 0.005 | 2.093(1.227,3.569) | 0.007 |
| Q3 | 1.469(0.865,2.494) | 0.155 | 1.597(0.947,2.692) | 0.079 |
| Q4 | 2.106(1.289,3.443) | 0.003 | 2.037(1.232,3.367) | 0.006 |

Model:Gender, age, race, education, PIR, somke, BMI, hypertension, hyperlipidemia were adjusted.
